# American Dental Convention

**Published:** 1863-08

**Authors:** 


### Proceedings of Societies.

### AMERICAN DENTAL CONVENTION,

### FIRST DAY—MORNING SESSION.

The Ninth Annual Meeting of the *American Dental Convention* assembled in White's Hall, Saratoga, on Tuesday, August 4th, 1863, and continued in session till Friday, the 7th.

The President and Vice President both being absent, the meeting was called to order by Dr. W. B. Roberts, Corresponding Secretary, who was appointed President *pro tem.*

The minutes of the last meeting were read.

The Treasurer reported one dollar as the required annual assessment.

The following members of the profession enrolled their names, and paid the annual fee:

New York.—B. T. Whitney, G. B. Snow, T. G. Lewis, Buffalo; L. W. Rogers, A. N. Priest, Thomas D. Evans, C. B. Foster, Utica; A. C. Hawes, Wm. H. Atkinson, J. H. Smith, W. A. Bronson, N. W. Kingsley, G. Chevalier, W. B. Roberts, F. H. Norton, A. Jones, E. A. L. Roberts, J. Allen, W. H. Dwinelle, New York; A. T. Smith, E. M. Skinner, Syracuse; Wm. C. Parks, Williamsburgh; D. S. Goldey, Oswego; S. B. Palmer, Tully; S. Mapes, Fishkill Landing; J. L. Clark, Waterloo; S. L. Smith, S. D. Arnold, Ballston Spa; J. A. Perkins, B. Wood, Albany; W. W. Perkins, Baldwinsville; J. G. Barber, Leroy; E. W. Sylvester, H. Jameson, Jr., Lyons; F. O. Hyatt, Cortland; L. W. Bristol, Lockport; A. M. Holmes, Morrisville; N. D. Bass, S. D. French, L. C. Wheeler, S. Andres, Troy; B. S. Burnham, Fort Edward; S. M. Robinson, Watertown; R. M. Howard, Knox Corners; G. W. Howell, Riverhead, L. I.; J. P. Beardsley, Clinton; E. L. Fuller, Peekskill; M. Tefft, Cambridge; J. A. Chase, Genesee; P. Harris, Skaneatles; H. A. Coe, Theresa; Geo. S. Allan, Newburgh; O. E. Hill, Brooklyn.—54.

Massachusetts.—T. Palmer, Fitchburgh; J. Fiske, Clinton; W. L. Bowdoin, Salem; O. F. Harris, H. F. Bishop, Geo. W. Newton, Worcester; F. Searle, Springfield; G. L. Cook, Milford; L. D. Shepard, Amherst; W. H. Jones, Northampton; H. M. Miller, Westfield.

Ohio.—C. R. Butler, Cleveland; Geo. Watt, Xenia; H. A. Smith, J. Taft, Cincinnati; C. Palmer, Warren; A. E. Lyman, Newton Falls; G. W. Keely, Oxford.

Connecticut.—W. W. Sheffield, New London; J. Woolworth, E. Strong, C. L. Smith, J. T. Metcalf, New Haven; T. S. Scranton, Madison; A. Hill, Norwalk; J. B. Snow, Bridgeport; J. A. Pelton, Middletown.

VERMONT.—H. H. Newton, St. Johnsbury; J. N. Scranton, Bennington; H. Kingsley, Middlebury; S. K. Thompson, Poultney; E. V. N. Harwood, Rutland.

PENNSYLVANIA.—T. L. Buckingham, C. N. Pierce, H. Townsend, O. Lund, G. W. Ellis, Philadelphia; J. B. Williams, Monongahela City.

NEW JERSEY.—A. W. Kingsley, Elizabeth; J. C. Robins, Jersey City.

RHODE ISLAND.—F. N. Seabury, Providence.

NEW HAMPSHIRE.—G. A. Young, E. G. Cummings, Concord.

WISCONSIN.—H. Faville, Milwaukee.

MICHIGAN.—H. Benedict, Detroit; J. A. Watling, Ypsilanti.

DELAWARE.—W. G. A. Bonville, Dover.

The Chairman announced that he had received a paper from the President, which was, on motion, accepted, and ordered to be read, at the appropriate time, as the retiring president's address.

The Treasurer reported a balance of $62.69 in the treasury. Messrs. Whitney, Rogers and Foster were appointed an auditing committee, who examined the Treasurer's report and found it correct. The report was accepted.

On motion of Dr. L. W. Rogers, Mr. Frank H. Norton was appointed reporter for the daily papers, at a salary not exceeding fifteen dollars.

On motion of Dr. W. H. Atkinson, 9 A. M. and 4 P. M. were fixed as the hours for meeting; and 1½ and 6½ P. M., as the hours for adjourning.

On motion of Dr. B. T. Whitney, the rules of business for last year were adopted for this.

Proceeded to the election of officers, T. L. Buckingham and N. W. Kingsley, tellers:

*President*, J. Taft, Cincinnati, O.; *Vice-President*, W. W. Sheffield, New London, Ct.; *Cor. Sec'y*, W. H. Atkinson, New York, N. Y.; *Rec. Sec'y*, C. N. Pierce, Philadelphia, Pa.; *Treasurer*, A. C. Hawes, New York, N. Y.

On motion, adjourned.

### FIRST DAY—AFTERNOON.

Met at 4 P. M. The officers elect were inaugurated, except the President, who was confined to his room by indisposition.

The paper from the retiring president was read by the president *pro tem.*

Proceeded to the regular discussions.

I. Causes influencing an abnormal development of the teeth.

The discussion was opened by Dr. SYLVESTER, who read a paper on the subject, giving as these causes: 1. Parental influence; 2. Improper diet; 3. Impure air; 4. Want of exercise.

Dr. SEARLE wished to know how and when the first case of irregularity occurred.

Dr. J. A. PERKINS had never seen the temporary teeth irregular.

Dr. ATKINSON had seen the temporaries irregular, but very rarely. He thought the paper just read erroneous in assuming that the American aborigines live according to nature. And he thought if the author had examined the skeletons of the western slopes he would have found defective teeth.

Dr. BURNHAM thought hereditary influence as clearly manifested on the teeth as on other organs.

Dr. HAWES called attention to one position of the paper which he regarded as peculiar. He had not seen the right side of the mouth like that of one parent and the left like that of the other.

Dr. Buckingham referred to a case in which the teeth of one side projected over, while the front teeth were even. The patient's mother and an aunt had similar defects, while other children in the same family copied the dental organs of the father. This hereditary influence is beyond our control in the human race. We breed horses with more care. Young folks often fancy each other on account of physical defects. American food is usually defective in tooth-making material; but this could be, to some extent, corrected by the use of the phosphates, which, as formerly prepared, were nearly insoluble, but are now improved in solubility. But the proper materials in the food were not all—they must be assimilated.

Dr. Searle said he was often asked by parents why their children's teeth are irregular. He was not able to tell. He regarded irregularity as accidental—as resulting from a cause we can not explain.

Dr. Whitney alluded to the effect of mixing races. The Germans have, usually, full, round mouths; the Irish, thin, sharp ones. When these races mix, the children's teeth are often irregular.

Dr. J. A. Perkins said that food had much to do with this subject. The Digger Indians, a race nearly toothless, subsist mainly on sour, bitter acorns.

Dr. Watt, by request, reported, to a limited extent, his success with subphosphate of lime, during the periods of uterogestation and lactation. He regarded the subphospate (bone phosphate) as the best of the phosphates for such cases. Though not very soluble in water, it is highly so in the fluids normally present in the stomach.

Dr. Williams thought we did not pay sufficient attention to the ill effects of the mother's breath on the babe. The babe is often forced to inhale, for a long period, its mother's breath instead of atmospheric air.

Dr. Ellis referred to the introduction of air through the mouth as injurious to the teeth.

Dr. Pierce referred to a case of a diseased mother with bad teeth, whose babes were born with defective enamel. If first dentition is defective, the second is usually so. He referred at some length to the effects of measles, scarlet fever, and whooping cough, on the permanent teeth in their formative stage, and suggested that children should be kept from these diseases till second dentition is well advanced.

Adjourned.

SECOND DAY—MORNING.

Called to order at 9 A. M., by the President *pro tem.*, who called the Vice-President to the chair, the President being still unable to appear. The minutes were read and approved.

Dr. W. B. Roberts offered the following resolution:

*Resolved*, That the Chair appoint a committee of five to make arrangements for the proper observance of the day of Thanksgiving appointed by the President of the United States. The Chair appointed W. B. Roberts, L. W. Rogers, N. W. Kingsley, G. Watt and W. H. Atkinson.

The committee on introducing dentists into the army was called on to report. Dr. Atkinson, the only member of the committee present, reported that but little had been done. On motion, resolved that a new committee be appointed.

Proceeded to regular discussions.

II. Treatment of dental irregularities, and appliances for the same.

Dr. N. W. Kingsley said he would talk a little about "appliances." Much of the machinery used in regulating teeth he regarded as too complex. He had seen appliances almost as complicated as a steam engine, which were said, by their manufacturers, to work "as perfectly as a lamb's tail," and he found they accomplished only about as much. When an incisor stands behind the arch, he places a gold band in front of the teeth, securing it at the ends to the molars or bicuspids, and puts a small rubber ring as a ligature around the tooth and over the gold band. If the tooth needs to be turned in its socket, he uses the same appliances, letting the band rest against the outer edge of the tooth. When necessary points can be soldered on the band to hold the ligatures so as to apply the force in the desired direction. Even if the upper teeth shut inside of the lower, the application is perfectly successful; for at night the teeth are not tightly closed, nor even in daylight, after the tooth becomes sore.

Dr. W. B. ROBERTS had used successfully the same appliances. Instead of half round wire, as suggested by Dr. Kingsley, he would make the band of plate, make a hole through the band, put a thread through the hole, and draw the rubber loop through it, after it is placed around the tooth. Sometimes the band would remain in place without clasps.

Dr. PALMER, of Mass., uses rubber ligatures. When the teeth are very irregular, take an impression and strike up a plate, let it pass outside where needed, and over the molars, to prevent occlusion of the mouth, and attach the ligatures where needed.

Dr. KINGSLEY, in answer to query, said that where one front tooth is out and another in, while the ligature draws one out the band presses the other in. He had seen an upper incisor that shut inside of the lower teeth drawn out to its place in three days.

Dr. ELLIS suggested that the flattening of the ends of the gold band, so that holes could be made through them, that the band might be secured to the molars or bicuspids by ligatures. He referred to cases which were readily corrected by the use of ligatures alone.

Dr. WOOLWORTH uses a rubber string, and weaves it back and forth among the teeth, when the general position of the arch is correct, while individual teeth are out of line.

Dr. WATT objected to rubber ligatures, on account of the constant pressure they kept up. He preferred something that would afford pressure up to a certain point, and then allow the parts to rest. For ligatures, he preferred flax thread. It contracts more, when moistened, than silk. The first time he was ever in Prof. Taylor's office, Prof. T. was correcting a bad case of irregularity by the simple appliance of flax ligatures. That was in 1852. In the use of rubber he thought there was danger of producing inflammation, and even sloughing. "Pressure and rest" is a law of nature not to be ignored in overcoming the resistance of living textures. This law is beautifully illustrated in the great mechanico-vital process which ushers us into responsible being. The same law is observed in the eruption of the dental organs, and, he thought, should not be ignored in changing their positions.

Dr. BUTLER did not believe with Dr. Watt, that nature works in paroxysms. He maintained that the teeth are developed by a constant process. When the laterals stand in and the cuspids out, he uses rubber ligatures.

Dr. E. A. L. ROBERTS uses rubber to change the position of the tooth, and thread to hold them in place.

Dr. BONVILLE exhibited a neat, ingenious apparatus, which is too complex for general adoption.

Dr. BUCKINGHAM, when the bicuspids are to be spread, uses a plate, and spreads the plate as the teeth yield.

Dr. W. B. ROBERTS said that, when the ten front teeth are irregular, he strikes a plate over the whole arch, leaving the plate open as far back as the irregularity extends, and by ligatures and other appliances, makes pressure in the desired direction.

At this point the President, Dr. TAFT, arrived, and was received with hearty cheers by the members of the Convention, who were all delighted to see that his health had improved to such extent that he could be present. The Vice-President appointed Drs. Hawes and Watt to conduct the President to the chair, who after being thus introduced, made a few brief and appropriate remarks, expressing his thankfulness for the honor conferred, and his regret that he was not able to discharge the duties of the office as he would like to do.

The discussions being resumed,

Dr. SEALE spoke of deformity from very prominent upper lip and jaw. A Russian nobleman thus afflicted, came to Paris to have the deformity corrected. The French surgeons declined to interfere. Some one told him there was a Yankee dentist there, and as Yankees were supposed to be especially ingenious, he called on him—the well known Dr. Brewster. Dr. B., by pressure, by means of a pad on the back of the head, and properly fitted straps, succeeded in correcting the features perfectly.

The Committee on Thanksgiving exercise presented a report, which was adopted.

On motion, 4½ o'clock, P. M., was appointed the hour for determining the place of the next meeting.

### III.—1. *Filling Teeth.* 2. *Filling Temporary Teeth.* 3. *Best Material for same.*

Dr. ROGERS would speak of best materials, and might surprise the Convention. Tin foil, as a general material, is better than gold. In many cases it was as good, in many better. In approximate cavities it was better than gold. He had seen many cases where it had proved better than gold in the same mouth. On account of its softness, it was objectionable on the grinding surfaces of the teeth. It was important to save teeth with cheap material if it can be done. (Dr. R. succeeded in surprising a good many of the members by these remarks, but made some subsequent statements, which gave the impression that he was misunderstood.)

Dr. J. A. PERKINS said we could learn something about filling yet. Last winter he had "blundered" on the use of the mallet, and could make fillings more solid than by hand. Dr. Wood's fusible, plastic metallic filling he regarded as better than tin.

Dr. SMITH said there was a lady now at Congress Hall who had worn a tin filling for thirty-four years. Gold fillings had failed in the same mouth.

Dr. HAWES would be very sorry if the impression should go abroad that this Convention considered tin as good as gold. The change of color he regarded as condemnatory.

Dr. ATKINSON said that, to feel the great importance of filling teeth, we must understand the causes which make it necessary. He is but a secondary plugger who only makes the tooth hold the filling. In first class work, the tooth holds the filling, and the filling holds the tooth. The indestructibility of gold was a strong argument in its favor. The manner, he said, was everything. Nothing could be more "stinking" than a soft gold filling. Every cavity should be saturate with pure creosote before inserting the gold. All soft tissue need not be taken out, provided this is done. The softened dentine in the bottom of the cavity acts as a good cap for the nerve, when combined with creosote.

SECOND DAY— AFTERNOON.

The Convention met at 4 o'clock, P. M. Vice-President in the chair. The same subject was continued.

Dr. WOOD read a paper on Plastic Metallic Filling.

Dr. PERKINS described a case in which a clasp tooth was broken off even with the gum, in which he drilled out the canal, screwed in a silver wire, filled around it with gold, and thus restored the office of the tooth.

The Chair now announced that the order of the day was the selection of the place for the next meeting, and a vote was taken, resulting in the choice of Philadelphia.

Resumed discussions.

Dr. WOOD demonstrated his method of using his plastic filling.

Dr. GOLDEY inquired if Dr. W.'s metal had the same objectionable qualities as the old fusible metal.

Dr. WOOD referred Dr. G. to the *Register* and *Cosmos*, where this question is examined at length.

Dr. ROGERS stated, in reference to contractility of alloys, that "mock iron" expands in congealing, and referred to the fact that the same property is claimed for Babbitt's metal.

Dr. W. B. ROBERTS was not familiar with the use of Wood's metal, would advise members to try it and report at the next meeting.

Dr. HILL said, (humorously), that Dr. W. had privately made a very important suggestion, that is, in large cavities, to put in a basis of "Hill's Stopping" before inserting the metal.

Dr. ATKINSON said that was a good idea. He liked Wood's metal, and gave practical suggestions as to its use.

Dr. PERKINS was in the habit of using Hill's Stopping, in large cavities, as a base, and Wood's metal on top of it. He thought the great fault with most dentists, is that they do not prepare themselves to do first class work.

Dr. BUTLER inquired if the alloy would do to work along with gold in a front tooth.

Dr. WOOD said galvanic action would result, if both were exposed.

Dr. WATT used no metal but gold for seven years. About a year ago, had filled a few teeth with Wood's alloy, and as many with tin. The alloy is doing better than the tin, and he expects to use it when cheap fillings are required.

Dr. C. PALMER approves of Wood's alloy. He would line the cavity with gold, in thin front teeth.

Dr. TEFFT had not had good results from two metals in the same tooth.

Dr. ECCLESTON suggested that tin fillings harden the dentine in contact thin them.

On motion, Cleft Palate, was made the order of the day for to-morrow, immediately after the thanksgiving exercises.

Dr. C. PALMER exhibited his instruments for preparing and filling fangs, and detailed his mode of operating.

THIRD DAY—MORNING.

Convention met at 9 A. M. The President in the chair.

The ministers expected to lead in the thanksgiving exercises not being present, the services were conducted under the supervision of the President.

By his request Dr. Watt read Psalm 107. Rev. Mr. Anderson, of Long Island, led in Prayer. Prof. Wood, and Drs. Rogers, Sheffield and W. W. Perkins led in singing that beautiful ode, "My country tis of thee," etc. Governor Cannon, of Delaware, was introduced by the President, and made some appropriate remarks on the state our country, and our duty as citizens, after which, Dr. Sylvester led in prayer, and was followed by remarks from Drs. Rogers, Watt, Atkinson. and Rev. Anderson. The Doxology was sung, Prof. Wood leading, and the apostolic benediction pronounced by Rev. Anderson, after which the Convention took a short recess. An hour and a half was spent in these special thanksgiving exercise, and the members generally, as well as the visitors present, including several ladies, were highly pleased with the appropriateness and earnestness of the services.

After a short recess the Convention was called to order. The minutes were read and approved.

The President annnouced the following as the Executive Committee:—L. W. Rogers, Utica, New York; A. W. Kingsley, Elizabeth, N. Jersey; J. A. Watling, Ypsilanti, Mich.; A. Hill, Norwalk, Conn.; H. A. Smith, Cincinnati, O.

The order of the day being "Cleft Palate," Dr. ATKINSON stated that the cause of Cleft Palate may be dynamical, chemical, or mechanical.

When an operation is practicable, the earlier it is performed the better. He would suggest the eighth day—circumcision day. In posterior cleft palate, where there is an abundance of the soft parts, cut them loose from the bone, make the edges perfectly fresh, and bring them together with wire sutures. In perforated palate, put in a plate to hold the dressings. When combined with harelip, use No. 6 needles to bring the lips together.

Dr. N. W. Kingsley explained the difference between voice and speech. Voice is natural—is formed in the larynx, and is not affected by cleft palate; while speech is acquired, and is greatly modified by cleft palate. A stronger reason for the operation of staphyloraphy, than the restoration of speech, sometimes exists. It happens, sometimes, that the child can not suck. The operation is not free from danger, and had even proved fatal. Speech, he maintained, is not usually restored by the operation. Dr. Valentine Mott, Jr., claims success in this direction; while Mott, Sr., disclaims perfect restoration of speech. The velum, after the operation, remains short and rigid. He preferred a properly constructed artificial velum to the operation, even when the latter is practicable. The best material for artificial palates, he found to be elastic vulcanized rubber. Gold, or other inflexible substances, will not answer. The first step in the process is to obtain a perfect impression of the parts, into the cleft, and to the posterior part of the pharynx. He now exhibited plaster models, of cleft palates, united in sectional blocks. He explained that the impression had to be taken in sections, and the models had to be molded on the same principle. A model of the velum is made of sheet gutta percha, or wax, and it must grasp the entire borders of the fissure, above and below, and extend back almost to the posterior wall of the pharynx. This is to be duplicated in hard rubber, which is to be smoothed and polished, applied to the plaster model, and corrected by warming and bending the margins, if necessary, and then one section of plaster mould is to be made, trimmed, varnished, and, by moulding in sand, a copy of it, in type metal, is to be made. The hard rubber pattern is to be placed in this, and another plaster section is to be made, which is also to be duplicated in type metal. The two metal sections are fitted together, including the pattern, and a third plaster section is to be made, to be followed by a fourth in like manner.

The artificial velum must be able to contract on itself, without wrinkling; and, to effect this, the posterior part of it must be made in laminæ This laminated structure is obtained by splitting the posterior part of the sheet of rubber, before vulcanizing, and laying in a small strip of writing paper, allowing it to extend as far forward as necessary. The paper prevents the adhesion of the two rubber surfaces. He uses rubber, prepared by an adept expressly for the purpose, and will aid any member of the profession in obtaining it. To prevent the laminæ from separating, so as to allow air to pass through, small semi-circular loops extend from one laminæ to another, the convexity of the loops extending upwards into the fissure; and to hold the velum in place, its anterior portion is somewhat thickened, and perforated, so as to admit a small gold cylinder, a small gold band is fitted to the arch of the mouth, and retained in place by clasps, and from this band a gold wire extends upward through the cylinder. The velum comes perfect from the mould, and is, therefore, ready for insertion, as soon as vulcanized, and by preserving the mould, can be duplicated as often as is desired. We are not able to explain on paper, the little internal arrangement of the mould by which the semi-circular loops are made, although it was explained by Dr. K. so as to be clearly understood by those who listened to him and saw his appliances.

By request, Dr. K. gave a more minute description of his mode of taking the impression. First, take an ordinary impression; and, with it as a guide, make a cup that will pass well up into the fissure, without spreading it, and extend far enough backward. Plaster, properly mixed, is to be pressed into the fissure, with a small spatula, or a narrow bladed table knife. This is allowed to harden somewhat, and the cup, prepared for the occasion, filled with plaster, is applied as for an ordinary impression. When the plaster in the cup has hardened sufficiently, the handle of the cup is suddenly depressed, and the plaster in it breaks loose from that in the fissure. The latter is now quite hard, and can be slipped backward, and caught with a properly shaped pliers, or tweezers, and removed from the mouth. The broken surfaces can be placed accurately together, and the impression is perfect.

Dr. K. exhibited a number and variety of duplicates of practical cases, and stated that in all these, the speech soon became perfect. He stated, further, that the irritability of the parts will not, in all cases, admit of a correct impression at first; but by handling, and repeated efforts, they became accustomed to the contact of foreign substances, and will tolerate the plaster.

Dr. DWINELLE endorsed the statements of Dr. K. as to the perfect restoration of speech.

Dr. J. ALLEN congratulated Dr. Kingsley, and the profession too. Here is an illustration of the advantages of *one ideaism.*

Dr. SEARLE inquired of Dr. K if the idea was original with him.

Dr. KINGSLEY said, not entirely. He had used the attainments already in existence, and progressed beyond them as far as he was able. He heard of an Englishman who wore an artificial palate, and on calling on him, found him to be Mr. Stearns, who had written on the subject. His instrument did not permit air to pass through the nostrils, and was, therefore, imperfect as a speech restorer.

Dr. HAWES moved that a vote of thanks be tendered to Dr. Kingsley for the very interesting and valuable description he has given us of his method of treating cleft palate. Motion carried unanimously. And, on motion of Dr. Hill, there was added to the resolution of Dr. Hawes, "and to him is due the honor of making the first perfectly practical artificial velum."

Dr. Hill said the lesson taught by this, is the success of patient and persevering study; and the importance of each one following his natural bent.

THIRD DAY.—AFTERNOON.

Met at 4 clock P. M. The consideration on cleft palate was closed; and "Filling Teeth" was declared in order.

Dr. C. Palmer demonstrated his mode of preparing and filling nerve canals. His instruments are very fine, and well adapted to the end aimed at. In tempering excavators, he hammers the steel cold, and is careful not to heat it too highly; and in drawing the temper, the color of the cutting edge should not change in the least.

IV.—*Diseases of the Antrum and Treatment.*

Dr. Atkinson remarked that the disease is the result of inflammation. When the pus is benign, simple puncture is all that is necessary. The cavity is lined with mucous membrane; and inflammation or engorgement there is not worse than catarrh, till the foramen that opens into the nostril is closed. The place to open it is between the fangs of the second bicuspid and first molar, as high up as the membrane will permit. The cavity can then be washed out, and whatever local remedy is indicated can be applied.

Dr. Newton had a second molar extracted, one root of which penetrated the floor of the antrum. Six weeks afterward, he found pus oozing out. A physician prescribed sulphate of zinc and rose water. The use of nitrate of silver effected a cure. The case occurred twelve years ago and has not returned.

Dr. Eccleston extracted a first bicuspid. Pus was discharged for two years. He extracted the second bicuspid and two molars, the roots of which were corroded. He made a large opening into the antrum, washed out with soap, and treated with a solution of nitrate of silver, 15 grains to the ounce. The recovery was perfect.

Dr. T. PALMER had a case in which the opening had a tendency to close. He inserted a gold tube, and washed with sulphate of zinc and hypochlorite of soda.

Dr. A. W. KINGSLEY reported the case of his father, in which the discharge was very offensive, accompanied with great loss of bone. He took wine and tonics, and washed with a solution of nitrate of silver, beginning with a weak solution and gradually increasing up to 30 grains to the ounce.

On motion, the order of business was suspended and the subject of NITROUS OXYD was taken up.

Dr. J. ALLEN regarded the oxyd as the best anæsthetic. He had tried all, and thought no other one worthy of the attention of dentists. Chloroform is not safe. Ether is a little better. Congelation is bad. Electricity is not reliable. The oxyd acts quickly. The patient is asleep in one or two minutes; and the effect lasts long enough to extract five or six front teeth, or two or three back ones.

Dr. SEARLE prefers the oxyd. Has used it daily for three weeks. He uses a mouth piece and a gas bag holding four gallons—empties the bag at the close of each operation. Its effect is not uniform. Patients can control themselves to a considerable extent. He had given it three times to a patient, to extract thirteen teeth.

Dr. S. S. WHITE said that commercial nitrate of ammonia is not sufficiently pure to be used for preparing the gas. A pure nitrate is to be used, and the gas should be passed through water.

Dr. BUCKINGHAM said the oxyd is certainly not as convenient as ether or chloroform. But is it better? He referred to the experiments of Davy with this agent, in 1800, and to the fact of its being used by Dr. Horace Wells. Its action he said, was very much like that of ether.

Dr. WATT had not used the oxyd. It was, certainly, less convenient than ether or chloroform. These he regarded as reasonably safe; in judicious hands, as safe as opium. It was not probable the oxyd would supersede the other anæsthetics; but if safer, it might answer a good purpose for trivial operations, on excessively timid patients. In such cases he did not use ether or chloroform.

Dr. ATKINSON called attention to the fact that chloroform has proved fatal only in light operations; the suggestion of Dr. W. was, therefore, good. He uses chloroform, and has had no bad results; but always feels an apprehension in giving it.

Dr. HILL began with ether, had no fatal, but some unpleasant cases. Gave chloroform three times—the last case so alarming that he abandoned it. He does not expect to use the oxyd.

Dr. W. B. ROBERTS offered the following:—

WHEREAS, This Convention, having for its object the elevation and advancement of our science, desires on all occasions to recognize, indorse, and to give encouragement to those of our numbers who contribute most largely to the progress and perfection of our noble art; and

*Whereas*, Dr. Norman W. Kingsley has this day presented and demonstrated to this Convention his peculiar method of restoring artificially the lost palate and velum, in a manner so clear and comprehensive as to entitle him to a substantial testimonial from this Convention; therefore

*Resolved*, That this Convention present to Dr. Kingsley a gold medal as an expression of their high appreciation of his valuable contribution to our profession.

*Resolved*, That a committee of five be appointed by the Chair to carry out the object of this resolution, with power to draw upon the Treasurer for an amount not exceeding fifty dollars. Carried.

On motion adjourned.

FOURTH DAY.—MORNING.

Met at 9 A. M. Minutes read and approved. Resumed regular discussions.

VI.—*Alveolar Abscess.*

Dr Atkinson spoke at some length on the pathology and treatment of alveolar abscess, giving the same sound views which we have formerly reported from his lips; and with which the readers of the *Register* are already familiar.

Dr. Dwinelle thought this matter altogether magnified. He might say "2 and 2 is 4, or that water is wet, and dry sand not quite so wet," but he would be telling nothing new. He happened to be a pioneer in the treatment of alveolar abscess. Thousands had cured alveolar abscess for twenty years back. Twenty-three years ago he could promise to cure nine out of ten cases; and he didn't think it necessary to make such ado about it. He gave a method of forcing creosote through the foramen in the fang. Fill the canal with creosote, and then press a gutta percha piston into it.

Dr. Roberts reported a case of abscess, apparently accompanied by mercurial salivation. The tooth had been filled with amalgam in 1842. Six years ago he took out the amalgam, cured the abscess, and filled by inserting a platinum wire in the canal, and building gold around it, to restore the shape of the tooth. The case is still doing well. This is the same case he reported at Niagara.

Dr. Buckingham thought the salivation was not caused by the amalgam, after so long a time. While on his feet, he wanted to protest against inconsiderate condemnation of new or modified practice.

Dr. Roberts had recently seen a tooth, the fang of which had been filled by Dr. Hudson, over forty five years ago.

Dr. Hawes did not agree with Dr. Dwinelle, that little or no progress had been recently made in the treatment of abscess. He thought much had been learned in the last year, even. If the treatment was so well understood twenty years ago, he was surprised that Harris hadn't heard of it.

The President announced the following as the committee on the appointment of dentists in the army: S. S. White, B. T. Whitney, and G. Watt.

VII.—*Mechanical Dentistry.*

Dr. Dwinelle referred to Dr. Holmes' method of preventing the sensation of heat in the mouths of patients wearing vulcanite. He simply drilled holes through the plates, and filled them with wire. The theory is that the wires conduct electricity. He had tested the practice, with good results, in three cases. The same result, he said, could be obtained by a metallic air chamber.

Dr. E. A. L. Roberts suggested that the wires gave relief by conducting heat, rather than electricity.

Dr. J. A. Perkins denounced vulcanite work at considerable length, without giving very *specific* objections.

Dr. Hawes, in many cases, preferred vulcanite to any thing else. He had looked this question squarely in the face for five years. Had seen but one or two patients who were not pleased with it after trial.

Dr. Whitney appreciated Dr. Holmes' suggestion. He thought the difficulty with vulcanite was caused by the air chamber. He preferred plates without chambers.

Dr. Goldey uses chambers for partial, but not full for sets.

Dr. W. B. Roberts was opposed to the use of vulcanite The best way to avoid difficulty with it was to use continuous gum work.

Dr. Butler said we must supply artificial teeth, because they are demanded. He would advocate no particular base, as the kind of base is less important than the style of executing the work. Had there been no necessity for vulcanite, it would not have been introduced.

Dr. Allen gave a brief history of continuous gum work; and compared its merits with those of gold and vulcanite. He thought vulcanite had a place to fill in mechanical dentistry. He suggested that the lower teeth, when the sockets are healthy, should be left in, and worked over without cutting them down. As an illustration of the advantages gained by this he exhibited his own teeth, and his mouth without them. This demonstration gave a clear idea of the practicability of this plan, and of the advantages gained by restoring the shrunken feature, than an hour's talk could have done; and for it, the doctor received, as he deserved, a hearty vote of thanks.

Dr. C. PALMER had no hobbies, but admired continuous gum. He enforced the importance of correct articulation. The face must be long enough; and in restoring the contour, the filling out must be done at the right place.

Dr. T. PALMER thought the burning sensation caused by vulcanite was owing to the closeness of the fit. He regarded continuous gum as the best work, but vulcanite fills a gap.

Dr. E. A. L. ROBERTS referred to the first dental vulcanizer, with its boiler large enough for an eight horse power engine, the chamber weighing about a ton, and requiring two men to lift on its cover, while a stout boy was kept busy at shoveling coals into the furnace. In August, 1860, he made the first single chamber vulcanizer, for which a patent was granted. He called attention to a new vulcanizer, which he thought an improvement on all before it.

Dr. GOLDEY wanted a "settled policy" in the construction of artificial teeth. Eighteen objections had been made to his own, and he presumed if these were corrected, there would still be as many suggested.

Dr. N. W. KINGSLEY said there could be no settled policy, as individual taste must govern in each case. And, in this matter of taste, lies the great difference among operators.

Dr. ROBINS, for trial plates in vulcanite work, uses the red wax on which he receives his artificial teeth.

Dr. WHITNEY makes sheet wax, for the same purpose, by combining five parts of white wax with one of venice turpentine, over a slow fire, and dipping a wet shingle into the melted mixture, often enough to make sheets of the desired thickness, when, by immersion in cold water, the sheets will be hardened, and may be separated from the shingle.

Dr. WILLIAMS uses waxed muslin, two or three thickenesses, for trial plates, and uses a combination of wax and sandarac to fasten the teeth to them for trial. If tin foil is used in vulcanizing, it can be readily removed from the plate by mercury.

The order of business was, on motion, suspended, to re consider the vote determining the place of the next meeting; and the result was the selection of Detroit, Mich. It was, on motion, resolved to adjourn, *sine die*, at 2½ P. M. to-day.

VIII.—*Miscellaneous Business.*

The President announced the following as the committee on the Kingsley medal: Drs. W. B. Roberts, A. C. Hawcs, John Allen, W. H. Atkinson, W. H. Dwinelle.

Dr. Atkinson read two papers, one on "Anomalous Cases," and the other on "Causes retarding Dental Progress," both of which we expect to lay before our readers.

The President made a short, earnest address on the subject of popular dental instruction, setting forth clearly its great importance to both operator and patient, and suggesting a variety of modes by which a cause so important may be advanced.

